# Altered Neurovascular Coupling in Unilateral Pulsatile Tinnitus

**DOI:** 10.3389/fnins.2021.791436

**Published:** 2022-01-21

**Authors:** Xiaoshuai Li, Ning Xu, Chihang Dai, Xuxu Meng, Xiaoyu Qiu, Heyu Ding, Rong Zeng, Han Lv, Pengfei Zhao, Zhenghan Yang, Shusheng Gong, Zhenchang Wang

**Affiliations:** ^1^Department of Radiology, Beijing Friendship Hospital, Capital Medical University, Beijing, China; ^2^Department of Otolaryngology Head and Neck Surgery, Beijing Friendship Hospital, Capital Medical University, Beijing, China

**Keywords:** cerebral blood flow, arterial spin labeling, regional homogeneity, functional magnetic resonance imaging, pulsatile tinnitus

## Abstract

**Objective:**

Altered cerebral blood flow (CBF) and regional homogeneity (ReHo) have been reported in pulsatile tinnitus (PT) patients. We aimed to explore regional neurovascular coupling changes in PT patients.

**Materials and Methods:**

Twenty-four right PT patients and 25 sex- and age-matched normal controls were included in this study. All subjects received arterial spin labeling imaging to measure CBF and functional MRI to compute ReHo. CBF/ReHo ratio was used to assess regional neurovascular coupling between the two groups. We also analyzed the correlation between CBF/ReHo ratio and clinical data from the PT patients.

**Results:**

PT patients exhibited increased CBF/ReHo ratio in left middle temporal gyrus and right angular gyrus than normal controls, and no decreased CBF/ReHo ratio was found. CBF/ReHo ratio in the left middle temporal gyrus of PT patients was positively correlated with Tinnitus Handicap Inventory score (*r* = 0.433, *p* = 0.035).

**Conclusion:**

These findings indicated that patients with PT exhibit abnormal neurovascular coupling, which provides new information for understanding the neuropathological mechanisms underlying PT.

## Introduction

Pulsatile tinnitus (PT) manifests as vascular somatosound synchronized with the pulse (Haraldsson et al., 2019). Sigmoid sinus wall anomalies are considered the most common and curable cause of PT (Dong et al., 2015; Mundada et al., 2015). Sounds and vibrations produced by abnormal hemodynamics in the venous sinus are perceived by the inner ear through the incomplete sinus wall (Li et al., 2021a). Sigmoid sinus wall reconstruction can effectively eliminate PT (Zhang et al., 2019). This disease state seriously affects patients’ daily lives, leading to irritability, anxiety, sleep disturbance, depression and even suicide (Li et al., 2020).

Recently, more attention has been given to the neuronal activity of patients with PT (Lv et al., 2015a,b, 2016b,^2017^). Spontaneous neuronal activity can be reflected by blood oxygen level-dependent (BOLD) signals in resting-state fMRI (Fox and Raichle, 2007). Lv et al. (2015a) used fMRI to measure altered amplitude of low-frequency fluctuation (ALFF) and regional homogeneity (ReHo) values in multiple brain regions of patients with unilateral PT, suggesting that abnormal brain activity existed in such patients. Subsequently, numerous studies also found abnormal functional connectivity between multiple brain regions and networks in these patients (Lv et al., 2015b,^2017^, ^2018^). The above studies indicated that there are neuropathological changes in PT patients.

According to the neurovascular coupling hypothesis, an increase in neuronal activity is accompanied by an increase in cerebral metabolic demand, leading to an increase in cerebral perfusion (Raichle and Mintun, 2006; Lanting et al., 2009; Venkat et al., 2016). Previous studies have used PET and SPECT to explore cerebral blood flow (CBF) changes in tinnitus patients, and found that there are altered CBF in multiple brain regions (Geven et al., 2014; Laureano et al., 2016). Perfusion and metabolism are tightly coupled in the brain (Aubert and Costalat, 2002). Several studies have found that the brain perfusion measured by arterial spin labeling (ASL) (Dai et al., 2009; Yoshiura et al., 2009; Wolk and Detre, 2012; Binnewijzend et al., 2013) has a good correlation with the brain metabolism measured by PET (Herholz et al., 2002; Du et al., 2006; Pagani et al., 2010; Bozoki et al., 2012) in the field of mild cognitive impairment and Alzheimer’s dementia. Due to its advantages of non-invasiveness, low cost and simplicity, ASL can be used to perform repeated studies on subjects. Thus, this technique has become a promising alternative technique and is widely used in various disease states (Haller et al., 2016). Recent studies have used ASL to identify CBF changes in multiple brain regions of PT patients (Li et al., 2020, 2021b).

However, the above studies were based on a single imaging technique to assess neuronal activity or cerebral perfusion in patients with PT, which cannot comprehensively reflect the neurovascular coupling disorder underlying this disease. Liang et al. combined BOLD and ASL techniques and found that neurovascular coupling reflects aspects of the underlying physiological function of the brain (Liang et al., 2013). Subsequently, some studies have found changed neurovascular coupling in the context of various diseases, confirming that it is related to the pathophysiological mechanism underlying the disease (Phillips et al., 2016; Tarantini et al., 2017; Zhu et al., 2017; Guo et al., 2018). To date, the neurovascular coupling status of PT patients remains unclear. In this study, vascular response was evaluated by CBF, and neuronal activity was calculated by ReHo. CBF/ReHo ratio was used to assess regional neurovascular coupling. We expect to understand the neuropathological changes underlying PT from a new perspective.

## Materials and Methods

### Participants

In this study, 24 right PT patients and 25 age- and sex-matched normal controls (NCs) were included. All patients showed pulse-synchronous noise (Li et al., 2021a), and sigmoid sinus wall dehiscence was considered the key etiology of PT by DSA and CTA/V. The exclusion criteria for all patients and NCs included hearing loss, MRI contraindications, hyperacusis, neuropsychiatric diseases, and history of head trauma. Tinnitus Handicap Inventory (THI) scores were exploited to assess PT severity. All participants signed written informed consent approved by the ethical committee.

### Data Acquisition

MRI data were acquired on a GE Discovery MR750 3.0 T scanner. The parameters for 3D pseudocontinuous ASL were as follows: repetition time (TR), 4854 ms; echo time (TE), 10.7 ms; slice thickness, 4 mm with no gap; in-plane resolution, 3.37 mm × 3.37 mm; number of excitations, 3; field of view (FOV), 240 mm × 240 mm; postlabel delay (PLD), 2025 ms; flip angle, 111°; and 36 slices. Resting-state BOLD imaging was obtained with the following parameters: TE, 35 mm; TR, 2000 ms; FOV, 240 × 240 mm; matrix, 64 × 64; flip angle, 90°; slice thickness, 4 mm with 1 mm gap; 200 time points; and 28 slices. During the scanning, all participants were asked to relax without thinking of anything, to remain motionless and awake, and to close their eyes.

### Cerebral Blood Flow Calculation

CBF maps were preprocessed using previously described methods (Li et al., 2021a,b). First, the CBF maps of 25 NCs were coregister to MNI space to generate a standard template using SPM8 software. Then, we registered the CBF maps from all participants to this standard MNI template. CBF maps were normalized by dividing the participant’s global mean CBF (Aslan and Lu, 2010). Finally, we smoothed the CBF maps using an 8 mm full-width at half-maximum (FWHM) Gaussian kernel.

### fMRI Data Preprocessing

BOLD images were preprocessed using Data Processing Assistant for Resting-State fMRI (DPARSF) software. To allow the signal to stabilize, the first 10 time points were removed from analysis. The specific processing steps included slice timing, realignment (head translation > 2.5° or motion > 2.5 mm were excluded), nuisance covariate regressions, filtering (0.01–0.08 Hz), and spatial normalization into MNI space with resampling to 3 × 3 × 3mm^3^.

ReHo was calculated using Kendall’s coefficient concordance of a given voxel with its twenty-six nearest neighboring voxels (Zang et al., 2004). Individual ReHo maps were divided by the global average ReHo value and then smoothed.

### Statistical Analysis

Statistical analysis was performed using SPSS v.22.0 software. We used two-sample *t*-test to investigate the difference in age and handedness between groups, and Fisher’s exact test was used to detect the difference in sex between groups (*P* < 0.05).

In CBF/ReHo ratio analysis, we used two-sample *t*-test to explore significant CBF/ReHo ratio differences between the NCs and PT patients, with gender and sex serving as nuisance covariates. Cluster-level false discovery rate (FDR) correction was used for multiple comparisons with *P* < 0.05. The same method was performed to explore group differences of ReHo and CBF. A correlation analysis (Pearson’s correlation) was performed to assess relationships between clinical data and altered CBF/ReHo ratio.

## Results

### Demographic Data

Table 1 shows the demographic data for all participants. The PT patients and NCs were well matched for sex (*p* = 0.667), age (*p* = 0.114), and handedness (*p* = 1.000). Across all patients, the THI score was 52.0 ± 23.6, and the PT duration was 34.5 ± 31.1 months.

**TABLE 1:** Demographic data for PT patients and NCs.

	PT (*n* = 24)	NC (*n* = 25)	P value
Age (years)	38.5 ± 9.9	34.0 ± 9.7	0.114^b^
Sex (male/female)	2/22	4/21	0.667^a^
Handedness	24 right-handed	25 right-handed	1.000^b^
PT duration (months)	34.5 ± 31.1	NA	NA
THI score	52.0 ± 23.6	NA	NA

*Data are presented as the mean ± standard deviation. PT: pulsatile tinnitus; NC: normal control; THI: Tinnitus Handicap Inventory; NA: not applicable.*

*^a^ Fisher’s exact test; ^b^ Two-sample t-test.*

### Altered Cerebral Blood Flow/Regional Homogeneity Ratio in Pulsatile Tinnitus Patients

**FIGURE 1:**
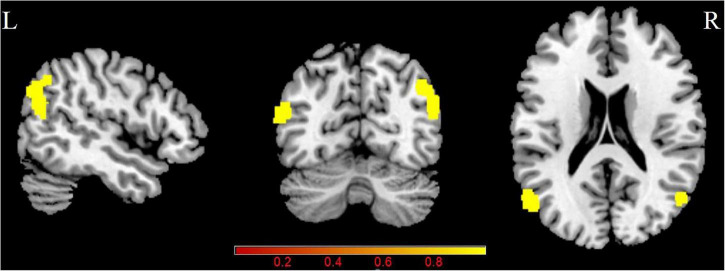
Group differences in CBF/ReHo ratio between patients with PT and NCs (*P* < 0.05, FDR corrected). PT: pulsatile tinnitus; NC: normal control; CBF: cerebral blood flow; ReHo: regional homogeneity.

Figure 1 and Table 2 shows the CBF/ReHo ratio difference between the two groups. The PT patients exhibited increased CBF/ReHo ratio in the right angular gyrus and left middle temporal gyrus than the NCs, and no decreased CBF/ReHo ratio was found in the PT patients (*p* < 0.05, FDR corrected).

**TABLE 2:** Brain regions with significant group differences in CBF/ReHo ratio.

Brain region	Peak MNI (mm)			Peak T value	Cluster size (mm^3^)
	x	y	z		
PT > NC					
R angular gyrus	52	−66	26	4.73	226
L middle temporal gyrus	−54	−68	18	5.06	118

*PT: pulsatile tinnitus; NC: normal control; CBF: cerebral blood flow; ReHo: regional homogeneity; MNI: Montreal Neurological Institute; L: left; R: right.*

### Altered Regional Homogeneity and Cerebral Blood Flow in Pulsatile Tinnitus Patients

**FIGURE 2:**
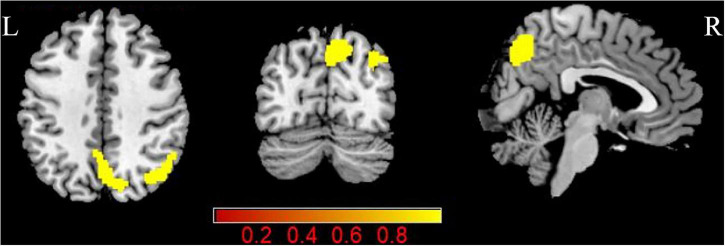
Group differences in CBF between patients with PT and NCs (*P* < 0.05, FDR corrected). PT: pulsatile tinnitus; NC: normal control; CBF: cerebral blood flow; ReHo: regional homogeneity.

Pulsatile tinnitus (PT) patients exhibited significantly increased CBF in the right angular gyrus and precuneus than the NCs (*p* < 0.05, FDR corrected) (Figure 2 and Table 3). There were no significant ReHo differences between the two groups.

**TABLE 3:** Brain regions with significant group differences in CBF.

Brain region	Peak MNI (mm)			Peak T value	Cluster size (mm^3^)
	x	y	z		
PT > NC					
R angular gyrus	46	−66	42	4.75	330
R precuneus	8	−72	48	4.62	345

*PT: pulsatile tinnitus; NC: normal control; CBF: cerebral blood flow; MNI: Montreal Neurological Institute; R: right.*

### Correlation Analyses

**FIGURE 3:**
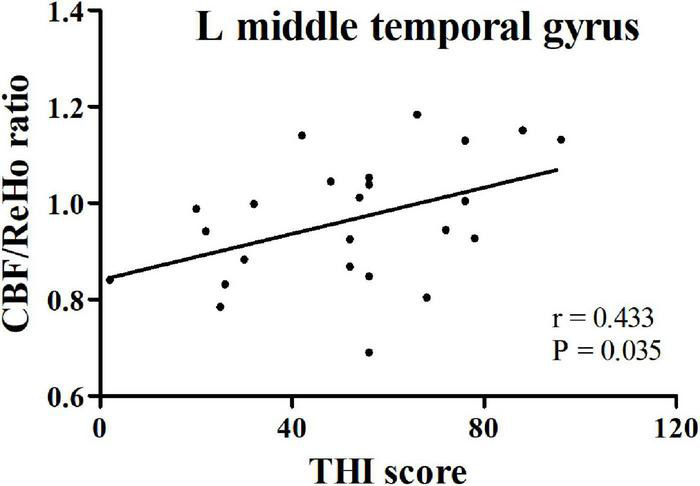
The correlation between CBF/ReHo ratio in the left middle temporal gyrus in PT patients and THI score. PT: pulsatile tinnitus; NC: normal control; CBF: cerebral blood flow; ReHo: regional homogeneity; THI: Tinnitus Handicap Inventory.

In the PT patients, increased CBF/ReHo ratio in the left middle temporal gyrus was positively correlated with THI scores (*r* = 0.433, *p* = 0.035) (Figure 3). We found no significant correlation between CBF/ReHo ratio in the right angular gyrus and clinical data.

## Discussion

This study investigated altered neurovascular coupling in PT patients by combining ASL and BOLD MRI. We found that patients with PT had increased CBF/ReHo ratio in the left middle temporal gyrus and right angular gyrus. Furthermore, the altered CBF/ReHo ratio in the left middle temporal gyrus was positively correlated with THI scores. These findings may help us understand the neuropathological mechanism underlying PT from the perspective of neurovascular coupling.

Previous studies have confirmed that CBF/ReHo ratio can offer more information on local neurovascular coupling alterations in diseases (Guo et al., 2018; Liu et al., 2021; Zhang et al., 2021). In a normal brain, CBF/ReHo ratio remains balanced. In PT, the deviation in the balance (i.e., abnormal neurovascular coupling) may lead to increases or decreases in CBF/ReHo ratio. Decreased CBF/ReHo ratio represents a relatively insufficient blood supply per unit of neuronal activity, while increased CBF/ReHo ratio represents a relative excess blood supply per unit of neuronal activity (Guo et al., 2018). The clinical correlation analysis found that CBF/ReHo ratio was related to the severity of PT, thus confirming the disruptive effect of altered CBF/ReHo ratio. More importantly, this ratio can identify abnormal brain areas with no obvious alterations in ReHo and CBF. Changes in CBF and ReHo in opposite directions may disrupt this balance. Therefore, slightly decreased ReHo and increased CBF may lead to significantly increased CBF/ReHo ratio in PT patients, while slightly increased ReHo and decreased CBF may lead to significant decrease in CBF/ReHo ratio (Guo et al., 2018). This mechanism can be used to explain why there were significant group differences in the CBF/ReHo ratio in these brain regions, but no significant differences were found in ReHo or CBF measurements. Thus, CBF/ReHo ratio can be used as a novel functional imaging index to evaluate neurovascular coupling alterations in disease.

Pulsatile tinnitus (PT) patients had increased CBF/ReHo ratio in the right angular gyrus than the NCs. Moreover, in the present study, increased CBF and normal ReHo were found in the right angular gyrus, suggesting that the increase in the CBF/ReHo ratio was mainly caused by increases in CBF. Prior studies showed that in patients with unilateral tinnitus, synchronized activity and connectivity within the gamma band were increased in the right angular gyrus as assessed using electroencephalography (Vanneste et al., 2011; Zhang et al., 2020). Chen et al. (2014) based on fMRI, found increased low-frequency fluctuations in chronic tinnitus patients, indicating increased neuronal activity in this region (Chen et al., 2014). A PET study also confirmed the increased angular gyrus activity in patients with chronic tinnitus (Song et al., 2012). This may be related to the function of the angular gyrus to participate in the integration of auditory stimuli, memory-related activities, self-awareness, and self-perception (Daselaar et al., 2006; De Ridder et al., 2014; Zhang et al., 2020). Moreover, the angular gyrus is involved in shifting the attention of chronic tinnitus patients from auditory phantom percept to visual cues in Heidelberg neuro-music therapy (Krick et al., 2017). Stimulating the hyperactive angular gyrus can eliminate tinnitus (Plewnia et al., 2007), indicating that there is a causal relationship between this area and tinnitus perception. In addition, this region is an important node in the dorsal auditory pathway, which converts auditory representations into premotor reactions (Karabanov et al., 2009). Schubotz et al. (2003) further found that coactivation of the superior premotor motor cortex and angular area is critical to the spatial positioning of auditory input (Schubotz et al., 2003). Note that the above studies included non-PT patients. Xu et al. used fMRI and found that the angular gyrus is participated in abnormal functional connectivity in PT patients (Xu et al., 2019), suggesting that this brain area plays a critical role in the pathophysiological mechanism underlying PT.

In addition, our preliminary results show that the CBF/ReHo ratio in the left middle temporal gyrus was higher in the PT patients than the NCs. The middle temporal gyrus is a part of the auditory association cortex (Verger et al., 2017), which is involved in auditory information processing. Animal studies have confirmed that tinnitus may be related to synaptic structure remodeling, enhanced synchronous and spontaneous neuronal activity in the auditory cortex (Adjamian et al., 2009; Henry et al., 2014). Voxel-based morphometry study has shown that tinnitus can cause significant cortical changes in the middle temporal gyrus [MNI coordinates (x, y, z):49, −70, 13; cluster size: 747] (Boyen et al., 2013). fMRI studies observed increased ALFF and ReHo in the middle temporal gyrus in tinnitus patients [MNI coordinates (x, y, z):60, −51, −9; cluster size:87] [MNI coordinates (x, y, z):60, −36, −3; cluster size:87] (Chen et al., 2014; Han et al., 2018), which also reflected abnormal regional functional changes in the auditory-related cortex in tinnitus patients. However, the above studies have focused on individuals without PT. Functional connectivity analysis conducted by Lv et al. (2016a) found that the middle temporal gyrus was involved in abnormal functional connectivity in PT [MNI coordinates (x, y, z):55, −52, 8; cluster size:79], thus confirming the vital role of this brain area. Wang et al. (2019) further found that functional connectivity in the middle temporal gyrus in PT patients changes over time, and its intensity can be used to quantitatively measure PT duration (Wang et al., 2019). These findings indicated that this brain area has important significance in the neuropathology underlying PT. In addition, the increased CBF/ReHo ratio in the left middle temporal gyrus were positively correlated with THI score in the PT patients, indicating the CBF/ReHo ratio in this region is more likely to reflect the severity of the disease.

This study has several limitations. First, we enrolled only right PT patients. In clinical work, right PT patients are more common than left PT patients (Eisenman et al., 2018) and may represent the general state of most patients. As the number of included patients increases, we will further explore the effect of PT side on neurovascular coupling changes. Second, the CBF/ReHo ratio is only an indirect reflection of neurovascular coupling. In the future, we expect that there will be direct neurovascular coupling indicators to describe the neuropathological mechanisms underlying PT. Third, this study found that the altered neurovascular coupling in the left middle temporal gyrus, which is a relatively large area. Thus, we have added MNI coordinates and cluster size of the middle temporal gyrus of other studies in the discussion section, and that found the location of this brain area is close to our result. Fourth, Changes in brain morphology may affect neuronal activity. Previous studies found that there was no significant difference in brain volume between PT patients and the NCs (Lv et al., 2015b, 2016a, 2017). Therefore, we did not carry out repeated morphological studies in this study. Fifth, this was a cross-sectional study. In the next step, we will explore changes in neurovascular coupling after PT is eliminated, and further explore the neuropathological changes associated with this condition.

## Conclusion

In conclusion, this study combined CBF and ReHo to describe regional neurovascular coupling changes in PT patients. Specifically, these patients exhibited increased CBF/ReHo ratio in the right angular gyrus and left middle temporal gyrus, and the altered CBF/ReHo ratio in the left middle temporal gyrus was positively correlated with the severity of PT. Our results provide potential imaging markers for understanding the neuropathological mechanism underlying PT.

## Data Availability Statement

The original contributions presented in the study are included in the article/Supplementary Material, further inquiries can be directed to the corresponding authors.

## Ethics Statement

The studies involving human participants were reviewed and approved by Ethics Committee of Beijing Friendship Hospital, Capital Medical University, Beijing, China. The patients/participants provided their written informed consent to participate in this study.

## Author Contributions

XL, NX, CD, XM, XQ, and RZ performed the experiment and collected, analyzed, or interpreted the data involved in the study. XL preprocessed image data, performed the statistical results, and drafted the manuscript. XL, PZ, HL, and ZW designed the study and ensured the questions related to all aspects of the work. PZ, HL, ZY, SG, and ZW gave critical comments on the manuscript. All authors contributed to the article and approved the submitted version.

## Conflict of Interest

The authors declare that the research was conducted in the absence of any commercial or financial relationships that could be construed as a potential conflict of interest.

## Publisher’s Note

All claims expressed in this article are solely those of the authors and do not necessarily represent those of their affiliated organizations, or those of the publisher, the editors and the reviewers. Any product that may be evaluated in this article, or claim that may be made by its manufacturer, is not guaranteed or endorsed by the publisher.
